# Research on interpretable machine learning modeling and spatial prediction of cross-landform landslide disaster mechanism: A case study of Shaanxi Province

**DOI:** 10.1371/journal.pone.0347996

**Published:** 2026-05-08

**Authors:** Mingming Han, Yiwei Yang

**Affiliations:** 1 College of Architecture and Civil Engineering, Xi’an University of Science and Technology, Xi’an, Shaanxi, China; 2 College of Safety Science and Engineering, Xi’an University of Science and Technology, Xi’an, Shaanxi, China; Leibniz University Hannover, GERMANY

## Abstract

The differential understanding of the landslidetriggering mechanisms across various geomorphic units is vital for enhancing regional disaster prevention. This study investigates the Loess Plateau area in northern Shaanxi and the Qinba Mountain Area in southern Shaanxi as the study areas, and constructs a 15dimensional evaluation factor system covering topography, geology, vegetation, and human activities. After eliminating collinearity factors via double tests of the Pearson correlation coefficient and variance inflation factor, Bayesian optimization is used to optimize hyperparameters for CatBoost, Random Forest, LightGBM, and XGBoost, and the SHAP framework is combined to perform global attribution, single-factor dependency analysis, and interaction-effect quantification. The results demonstrate that CatBoost performs best across both geomorphic areas, achieving AUCs of 0.8307 and 0.8252 in the test sets. In northern Shaanxi, a discrete patch pattern driven by “human-water” coupling is observed, with population density and the topographic moisture index contributing 37.2%. In contrast, Southern Shaanxi exhibits a continuous, band-shaped distribution controlled by “structure-topography” is observed, and elevation and lithology dominate the model’s decision-making. The SHAP-dependent map identified cross-geomorphic differences in the population density threshold of 99.83 people/km^2^, the slope threshold of 2.21° in northern Shaanxi, and the elevation threshold of 1194.73m in southern Shaanxi. Furthermore, the interaction network revealed an antagonistic effect of DEM and POP in northern Shaanxi and of DEM and LULC in southern Shaanxi. The framework proposed a quantitative, explainable basis for differentiated disaster prevention strategies in this research.

## Introduction

Landslides, as extremely destructive geological disasters worldwide, seriously threaten human life and property safety as well as regional sustainable development [1, 2]. China is among the countries most affected by landslides worldwide. According to the National Bureau of Statistics (http://www.stats.gov.cn), there were about 243,412 landslide disasters across the country from 2004 to 2024, causing about 24,000 casualties. Landslide disasters account for a significant proportion of the total number of geological disasters in the country [3, 4]. Against this severe backdrop, accurately identifying areas prone to landslides and elucidating their mechanisms of disaster-causing is not only an urgent need to enhance capacity for geological disaster prevention and control, but also a key scientific issue for ensuring urban and rural safety and the construction of ecological civilization [5]. China has a vast territory and a complex and diverse geological environment. The mechanisms that cause landslides in different geomorphic units (such as the Loess Plateau and rocky mountainous areas) differ significantly. Traditional research often treats regions as homogeneous wholes, ignoring the controlling role of geomorphic heterogeneity in the logic of disasters, thereby making it challenging to guide differentiated disaster-prevention practices precisely. Therefore, constructing a cross-geomorphic landslide-susceptibility evaluation framework and revealing differences in disaster-causing mechanisms across different geological backgrounds is of great strategic significance for enhancing the pertinence and scientific nature of regional disaster prevention and control [6]. Shaanxi Province is located in the inland hinterland of China, spanning the Yellow River and the Yangtze River basins from north to south. From north to south, it successively transitions into three central geomorphic units: the Loess Plateau in northern Shaanxi, the Guanzhong Plain, and the Qinba Mountains in southern Shaanxi. It is an ideal area for studying the spatial heterogeneity of landslide disasters. Among them, there are significant differences in material composition, geomorphic processes, and dynamic environments between the Loess Plateau area in northern Shaanxi (covered by Quaternary aeolian loess) and the Qinba Mountain Area in southern Shaanxi (a rocky mountainous area), providing a natural experimental field for cross-geomorphic comparative studies. Notably, Northern Shaanxi is characterized by a high frequency of loess landslides often linked to intensive human disturbances, while Southern Shaanxi suffers from more intense, rainfall-triggered disasters in complex rocky terrains that often result in significant casualties and socio-economic losses.

Landslide Susceptibility Mapping (LSM) is a method for quantitatively predicting the spatial probability of landslide occurrence by comprehensively considering multiple source characteristics, including regional topography and geomorphology, geological structure, hydro-meteorology, and human activities. It has significant strategic significance for regional disaster risk management and territorial space planning [7–9]. Early LSM research mainly used traditional statistical methods, such as the information volume model [10], the frequency ratio method [11], and linear regression [12]. These models, based on the statistical relationship between historical landslide data and the disaster-forming environment, offer strong quantitative analysis capabilities and objective results. However, the relationship between landslides across different regions and their inducing factors is highly complex and nonlinear [13]. Traditional statistical models perform poorly at characterizing this highly nonlinear mechanism that causes disasters and are often subjective when determining factor weights, making it difficult to efficiently process massive, high-dimensional raster data spanning multiple dimensions such as topography, geology, vegetation, and human activities. Its evaluation accuracy also falls short of the current refined disaster early warning’s actual needs.

With the rapid development of data mining technology, machine learning (ML) methods have been widely applied in the field of landslide susceptibility mapping due to their outstanding nonlinear fitting capabilities [14], covering various algorithms such as Support Vector Machine (SVM) [15], Decision Tree (DT) [16], and Random Forest (RF) [17, 18]. Machine learning can deeply explore the intrinsic connections between historical landslide sites and complex disaster environments, demonstrating significant advantages in evaluation accuracy, generalization performance, and overfitting suppression. Pradhan et al. [19] confirmed, through comparative studies, that machine learning models exhibit outstanding performance in capturing the association between environmental factors and landslides; Liao et al. [20] explored the robustness of the model across different grid resolutions to identify potential conditioning factors and enhance prediction performance. In recent years, the Gradient Boosting algorithm family has attracted significant attention for its powerful predictive performance and computational stability. Especially the introduction of algorithms such as XGBoost, LightGBM and CatBoost has provided efficient means for processing massive raster data. Xu et al. [21] applied ensemble learning to landslide prediction, effectively enhancing the model’s robustness and generalization. However, most existing studies treat the research area as a homogeneous whole, ignoring the significant spatial heterogeneity of disaster-causing mechanisms in complex geological environments, thereby making it difficult to accurately identify the key disaster-causing factors and their action patterns across different geomorphic regions.

Although machine learning models far exceed traditional statistical models in evaluation accuracy, their complex “black box” characteristics lead to low transparency and credibility in the decision-making process, making it difficult to intuitively explain to decision-makers the contribution logic of various environmental factors to landslide triggering. This seriously limits their in-depth application in high-risk geological disaster prevention and control. It also hinders its evolution from a purely data-driven to a physical-mechanism-driven approach. In recent years, Post-hoc Interpretation algorithms have provided a new path to break through model black boxes [22–24]. Among them, Shapley additive interpretation (SHAP) [25], with its solid game-theoretic foundation, operational convenience, and comprehensive interpretability from the global to the local, has gained extensive attention in the academic community. Zhou et al. [26] proposed an interpretable combined model based on SHAP and XGBoost, providing a scientific tool with both accuracy and transparency for disaster prevention and control. However, the application of SHAP in the field of landslides mainly focuses on a single geomorphic type, lacking a systematic deconstruction of the differences in disaster-causing mechanisms under different geomorphic backgrounds, and thus fails to answer the key scientific question of “how the same factor plays a differentiated role in different geological environments” [27].

Geomorphic features are the core components of the Earth’s surface system. The geomorphic classification system not only defines the physical properties of the surface from the aspects of geometric elements (topography, elevation, slope, etc.) and constituent materials (Quaternary loose loess and bedrock), but also reveals the internal and external forces that shape the geomorphic form and their causal environment [28]. Under different geomorphic backgrounds, the fundamental differences in geological structure, environmental background, and dynamic logic lead to entirely different disaster-causing responses. However, previous studies often regarded the entire region as a single evaluation unit. This approach is practical in local areas with uniform terrain. However, it has significant limitations when dealing with regions with large spans and complex environments [29–31], making it difficult to accurately measure the local contributions of various condition factors under different backgrounds. It is impossible to identify the inherent dynamic laws governing landslide occurrence across different geomorphic types.

In response to the above research gaps, this paper selects the Loess Plateau area in northern Shaanxi and the Qinba Mountain Area in southern Shaanxi, which have significantly different geological conditions within Shaanxi Province, as typical comparison areas, and constructs a cross-geomorphic research framework of “modelling - comparison - interpretation”. The specific research contents include: (1) The Bayesian optimization algorithm is utilized to automatically optimize the hyperparameters of CatBoost, RF, XGBoost, and LightGBM, and construct the landslide vulnerability evaluation models for different geomorphic units. Through comparative analysis, the optimal model is selected to generate high-precision zoning maps, thereby verifying the generalization performance of the machine learning model across cross-geomorphic environments. (2) Introduce the SHAP interpretation framework. Using a global importance ranking and a single-factor dependence graph, deconstruct and compare the driving factors and physical thresholds of the dominant landslides in the loess region and the bedrock mountain area, and reveal the differentiated laws governing cross-geomorphic mechanisms of disaster causation. (3) Utilize the interaction values of SHAP to quantitatively analyze the coupling and synergy effects among the dominant factors and clarify the nonlinear coupling mechanism of landslides causing disasters under different geomorphic backgrounds. This study aims to overcome the limitations of traditional “black box” modelling and “homogeneous” evaluation, and to construct a landslide vulnerability evaluation method system that is highly accurate, highly interpretable, and highly adaptable to landforms, providing a scientific basis for differentiated disaster early warning in complex geological environments.

## Materials and methods

### 1. Overview of the study area

**Fig 1:**
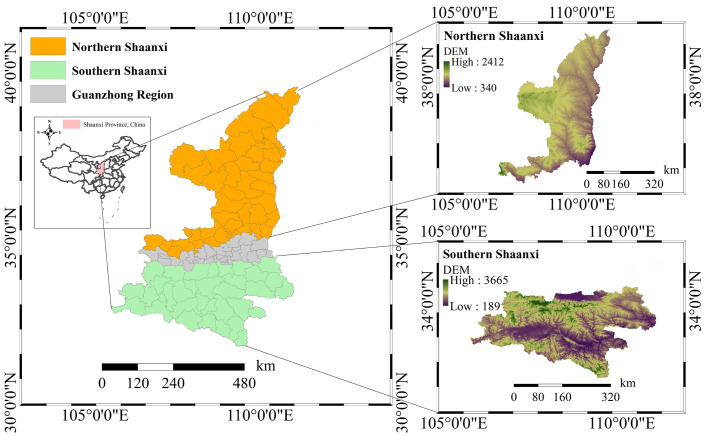
Study area. Geomorphological regions of Shaanxi: Northern, Guanzhong, and Southern. Right panels show DEM data for the study areas in Northern and Southern Shaanxi.

Shaanxi Province is located in the inland hinterland of China, spanning the Yellow River and the Yangtze River basins. Influenced by the unique geographic span between the north and the south, the province’s natural environment exhibits significant geomorphic differentiation. This study selects the Loess Plateau area in northern Shaanxi and the Qinling-Ba Mountain Range area in southern Shaanxi as typical research objects. The Loess Plateau area in northern Shaanxi is located in the northern part of Shaanxi Province, and its surface is widely covered by a thick Quaternary aeolian loess layer (usually over 100 meters thick). The landform types in this area are mainly loess mounds, loess ridges and dense gully systems, with fragmented terrain. Loess materials possess engineering geological characteristics such as high porosity, well-developed vertical joints and strong water sensitivity. The primary type of landslide in this area is a loess landslide. Intense human engineering activities (cutting slopes for road construction and house building) are important triggering factors, forming a typical “human-land” contradiction. The Qinba Mountain Area lies in southern Shaanxi Province. It includes two major mountain ranges: the Qinling Mountains and the Bashan Mountains. It is a typical high-mountain, deep-canyon bedrock landform. The terrain in the area undulates sharply, with a significant relative height difference. The lithological composition of the bedrock mainly controls the stability of the slope, the degree of development of structural planes, fault tectonic activity, and the effects of heavy precipitation. This area has a north subtropical monsoon climate with abundant, highly concentrated precipitation, which is highly likely to trigger a chain of mass shallow landslides, collapses, and debris flows. The geographical location and geomorphic zoning characteristics of the study area are shown in Fig 1.

### 2. Data sources and data processing

#### 2.1 Data source.

The fragile, disaster-prone environment controls the occurrence of landslides. Based on careful consideration of the geographical heterogeneity between northern and southern Shaanxi, this study selected 15 initial evaluation factors across four dimensions: topography, geology, vegetation, and human activities (Table 1). To accurately capture the different disaster-causing patterns in the two regions, based on general factors, region-specific selected factors were set for northern and southern Shaanxi. In the northern Shaanxi region, the distance from the road (DTR) and population density (POP) were selected to quantify the disturbance effect of highintensity human engineering activities on the stability of loess slopes. The topographic position index (TPI) and topographic moisture index (TWI) were selected to describe the spatial heterogeneity and water sensitivity characteristics of the loess ridge and mound landforms. In the southern Shaanxi region, the distance from the fault (DTF) and Lithology were selected to reflect the inherent constraints imposed by the fault structure and the complex bedrock composition of the Qinling orogenic belt on slope stability. The terrain undulation (RDLS) was selected to describe the driving effect of the difference in gravitational potential energy of high mountain and deep canyon landforms on the occurrence of landslides. In addition, elevation (DEM), Slope (Slope), Aspect (Aspect), Profile curve (Profile curve), Plan curve (Plan curve), distance from river (DTW), normalized vegetation Index (NDVI), and land use type (LULC) are used as general factors. To construct the basic topographic, vegetation, and hydrological background constraints of the two places. The study treats landslide susceptibility as a binary classification problem, and the sample data are sourced from the Resources and Environmental Science Data Platform (https://www.resdc.cn/). To enhance the model’s classification robustness, this study randomly selected equal numbers of negative sample points and positive samples from the non-landslide area, at a 1:1 ratio.

**Table 1:** Evaluation factors and data sources affecting landslides in the study area.

Category	Evaluation factor	Abbreviation	Region	Data source
Human activities	Distance from the road Population density	DTR POP	Northern Shaanxi Northern Shaanxi	(http://www.webmap.cn/)
Topography and landforms	Terrain position index Terrain moisture index Terrain undulation	TPI TWI RDLS	Northern Shaanxi Northern Shaanxi Southern Shaanxi	Geospatial cloud data (SRTM)
Geological background	Lithology Distance from the fault	Litholo DTF	Southern Shaanxi Southern Shaanxi	(https://zenodo.org/record/52109)
Basic characteristics	Elevation, slope, aspect, profile curvature, plane curvature, vegetation index, land use, distance from the river	DEM、Slope、Aspect、Profile curve、Plan curve、NDVI、LULC、DTW	Northern Shaanxi、Southern Shaanxi	(http://www.webmap.cn/)

#### 2.2 Data preprocessing and raster unification.

As the 15 evaluation factors selected in this study originated from multiple channels such as satellite remote sensing, ground investigation and meteorological monitoring, there are significant differences in their original formats, spatial resolutions and dimensional units. To ensure that the machine learning model can accurately extract features, this study implemented a strict preprocessing procedure on the original raster data. Taking the DEM with a resolution of 30 m as the benchmark, all raster data are uniformly resampled to 30 m × 30 m by using the bilinear interpolation method (continuous factor) and the nearest neighbor method (classification factor). All layers are uniformly projected onto the WGS_1984_UTM_Zone_48N coordinate system. The continuous factor values were mapped to the [0, 1] interval by using Min-Max Normalization [32] to eliminate the influence of dimensional differences on the convergence speed of the model. The formula is as follows:


$$X_{norm}=\frac{X-X_{min}}{X_{max}-X_{min}}$$ (1)

Where ${\text{X}}_{\text{norm}}$ is the normalized factor value, X is the original pixel value, and ${\text{X}}_{\text{max}}$ and ${\text{X}}_{\text{min}}$ are the maximum and minimum values of this factor in the study area, respectively.

**Fig 2:**
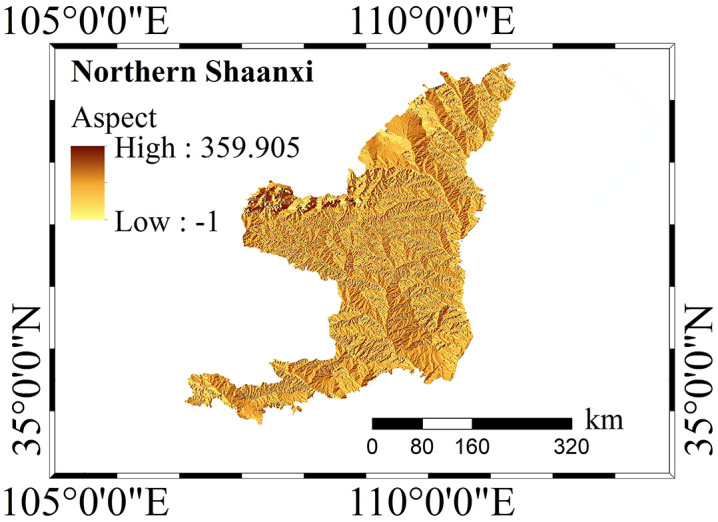
Evaluation factor map of Northern Shaanxi. The map reveals the spatial distribution characteristics of the aspect factor in the loess hilly-gully regions of Northern Shaanxi, with spatial attributes extracted from a high-resolution Digital Elevation Model.

**Fig 3:**
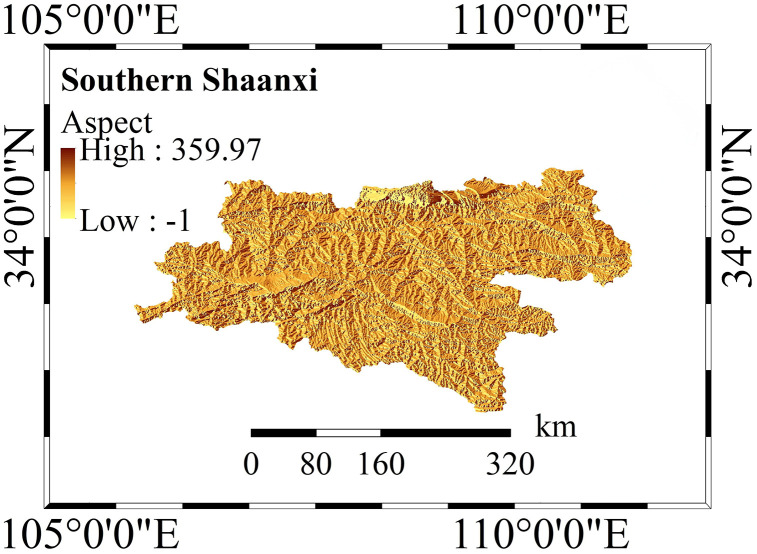
Evaluation Factor Map of Southern Shaanxi. The map reveals the spatial distribution characteristics of the aspect factor in the mountainous landscapes of Southern Shaanxi, with spatial attributes extracted from a high-resolution Digital Elevation Model.

As cross-regional research involves a large amount of edge data, this study masked all raster layers. The administrative boundaries between northern and southern Shaanxi were strictly cropped, and invalid values in all layers were eliminated or averaged filled to ensure that there were no null values in the data matrix input into the model. Subsequently, the processed evaluation factor data were visualized (Figs 2 and 3). This ensures the stability of the CatBoost, RF, XGBoost, and LightGBM algorithms in this study during training.

#### 2.3 Factor correlation and multicollinearity analysis.

**Fig 4:**
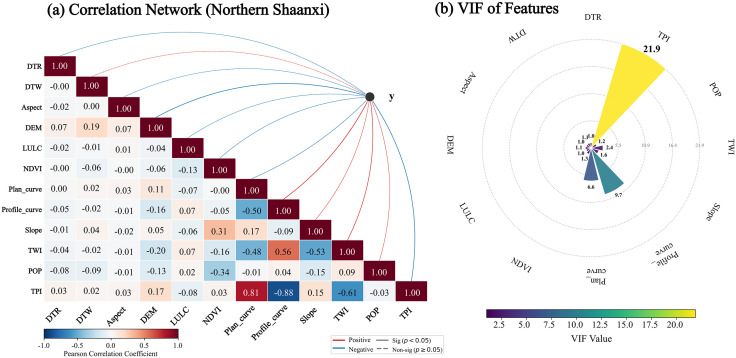
Factor Correlation and Multicollinearity Analysis in Northern Shaanxi Region. **(a)** The sub-figure combines a Pearson correlation matrix with a topological network to quantitatively reveal the interconnections among evaluation factors and their nature of influence on the target variable (red lines for positive and blue lines for negative correlation). **(b)** The radial plot further evaluates the multicollinearity of input features, ensuring the robustness of the predictive model by calculating the VIF values of each factor.

**Fig 5:**
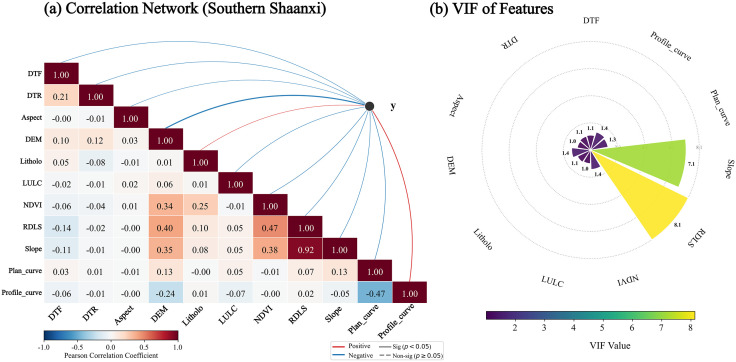
Factor Correlation and Multicollinearity Analysis in Southern Shaanxi Region. **(a)** The sub-figure combines a Pearson correlation matrix with a topological network to quantitatively reveal the interconnections among evaluation factors and their nature of influence on the target variable (red lines for positive and blue lines for negative correlation). **(b)** The radial plot further evaluates the multicollinearity of input features, ensuring the robustness of the predictive model by calculating the VIF values of each factor.

Before constructing a landslide susceptibility model, it is necessary to assess the independence among the evaluation factors. In this study, the Pearson correlation coefficient and the variance inflation factor (VIF) were introduced for double testing [33, 34]. For the loess region of northern Shaanxi, the Pearson correlation matrix is shown in Fig 4a. The results show that the factors such as population density (POP), distance from road (DTR), and topographic position index (TPI) selected for northern Shaanxi are mostly at a weak correlation level with the fundamental topographic factors. However, in additional VIF tests (Fig 4b), it was found that the VIF for the topographic potential Index (TPI) exceeded 10, indicating a strong linear dependence on slope and other micro-geomorphic indicators. To ensure the robustness of the research algorithm model, TPI was excluded from the characteristic factors of northern Shaanxi in this study. The final retained factor VIFs were all less than 10, ensuring the independence of the feature set for northern Shaanxi. For the Qinba Mountain Area in southern Shaanxi, the Pearson correlation matrix is shown in Fig 5a. Analysis shows that the correlation coefficients among factors such as Lithology (Litholo), distance from fault (DTF), and topographic undulations (RDLS) selected in southern Shaanxi are all below 0.6, indicating strong spatial independence. The VIF test results further confirmed (Fig 5b) that the VIF values for all selected evaluation factors in the southern Shaanxi region ranged from 1.12 to 3.58, well below the collinearity threshold. This indicates that the characteristic system of southern Shaanxi can reflect the structural and topographic features of the bedrock mountainous area in multiple dimensions, without collinearity.

### 3. Model construction

#### 3.1 Bayesian optimization.

To avoid subjectivity and the high computational cost of manual parameter adjustment, Bayesian optimization is adopted to perform automatic hyperparameter search. This method achieves an approximate optimal solution with fewer iterations by constructing a probabilistic proxy model of the objective function and using an acquisition function to balance global search and local exploration [35]. Its basic form is:


$$P\left(A|B\right)=\frac{P\left(B|A\right)P(A)}{P(B)}$$ (2)

Where, P(A/B) represents the Posterior probability, that is, the probability distribution of parameter A when the observed data B is known. P(B/A) represents the Likelihood function, indicating the possibility of the occurrence of observed data B under A specific parameter a. P(A) represents the Prior probability, that is, the initial understanding of parameter A. P(B) represents marginal Evidence and serves as a normalizing constant.

#### 3.2 Integrative learning model construction.

To improve the robustness and generalization of landslide prediction, four mainstream ensemble learning algorithms were selected for comparison: CatBoost, Random Forest, LightGBM, and XGBoost. Each model takes the decision tree as the base learner and enhances the nonlinear fitting ability through the integration strategy [36, 37, 38].

##### 3.2.1 CatBoost.

CatBoost uses an ordered boosting strategy to reduce gradient bias and can handle categorical features directly. The model form is an additive model:


$$F(x)=\sum_{t=\text{1}}^{T}\gamma_{t}h_{t}(x)$$ (3)

As an additive model, the final prediction function F(x) of CatBoost is composed of multiple fundamental weak learners $h_{t}$(x) superimposed, where T is the number of iterations and $\gamma_{t}$ is the weight of the t-th tree. In each iteration, the model fits the residuals of the previous round by minimising the loss function L.

##### 3.2.2 Random forest.

Random forests build multiple decision trees using the Bagging mechanism and reduce variance through feature random subsampling. The prediction result is the average output of each sub-model:


$$H(x)=\frac{\text{1}}{k}\sum_{i=\text{1}}^{k}h_{i}(x)$$ (4)

Among them, H(x) is the probability value of the model’s landslide-susceptibility output. k is the total number of decision trees in the random forest. $h_{i}(x)$ is the prediction result of the i-th decision tree based on the input environmental factor.

##### 3.2.3 *LightGBM.*

LightGBM adopts a gradient-unilateral sampling and feature bundling strategy to reduce computational complexity and improve fitting efficiency through leaf growth patterns. Its optimization objective is:


$$L^{\left(t\right)}=\sum_{i=\text{1}}^{n}l\left(y_{i},\overset{\left(t-\text{1}\right)}{\underset{i}{\hat{y}}}+h_{t}(x_{i})\right)+\Omega (h_{t})$$ (5)

Where l represents the Loss function, which measures the difference between the predicted and actual values, $h_{t}(x_{i})$ is the output of the t-th regression decision tree. $\Omega (h_{t})$ is the Regularisation term, which is used to control the complexity of the model and prevent overfitting.

##### 3.2.4 *XGBoost.*

XGBoost introduces second-order gradient information and complexity penalty terms in the objective function to enhance the convergence speed and suppress overfitting:


$$Obj^{\left(t\right)}=\sum_{i=\text{1}}^{n}l\left(y_{i},\overset{\left(t-\text{1}\right)}{\underset{i}{\hat{y}}}+f_{t}(x_{i})\right)+\Omega (f_{t})$$ (6)

Where l is the loss function, which measures the difference between the predicted value $\hat{y}$ and the true value $y_{i}$, $f_{t}(x_{i})$ is the regression decision tree generated in the t-th iteration. The regularization penalty term $\Omega (f_{t})$ is defined as $\Omega (f)=\gamma T+\frac{\text{1}}{\text{2}}\lambda ∥w{∥}^{\text{2}}$,which is used to limit the number of leaf nodes *T* and the leaf weights *w*.

#### 3.3 SHAP framework.

To reveal the model prediction mechanism, the SHAP method is adopted for feature attribution analysis. This method is based on the Shapley value theory, decomposing the prediction results into the sum of each factor’s contributions to provide explanations at both the global and individual levels. Its expression form is:


$$f(x)=\phi_{\text{0}}+\sum_{i=\text{1}}^{M}\phi_{i}\overset{\prime }{\underset{i}{z}}$$ (7)

Among them: f(x): The final predicted output of the model (i.e., the probability value of the landslide occurrence). $\phi_{\text{0}}$: Base Value, that is, the average of the predicted values of all samples. M: The total number of input evaluation factors. $\phi_{i}$: The SHAP value (contribution value) of the i-th factor. If $\phi_{i}>\text{0}$, it indicates that this factor promotes the occurrence of landslides; Conversely, it inhibits the occurrence of landslides. $\overset{\prime }{\underset{i}{z}}$: A binary variable indicating whether a feature exists (usually 1 in landslide modelling).

## Results

### 4. Optimization and accuracy comparison of cross-terrain models

#### 4.1 Model selection for the northern Shaanxi region.

To verify the applicability of various machine learning algorithms in the Loess Plateau region of northern Shaanxi, this study randomly divided the landslide sample dataset into a 70% training set and a 30% test set. By comparing the ROC curve performances of four models, namely Random Forest, XGBoost, LightGBM and CatBoost, on two sets of datasets, the final prediction scheme was determined.

**Fig 6:**
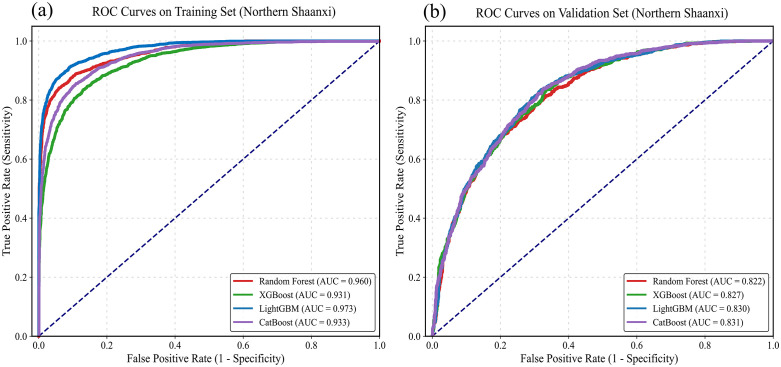
Augmentation values of ROC curves in Northern Shaanxi. (a) training set; (b) test set.

During the training phase, as shown in Fig 6a and Table 2, all four models demonstrated extremely high fitting accuracy. The AUC values for Random Forest, XGBoost, LightGBM, and CatBoost were 0.960, 0.931, 0.973, and 0.933, respectively. All four algorithm models have demonstrated their powerful learning capabilities for the characteristics of complex loess disasters. However, during testing, the performance of each model varied across test set samples that were not used in model construction. The AUC values for Random Forest, XGBoost, LightGBM, and CatBoost are 0.822, 0.827, 0.830, and 0.831, respectively. The differences between it and the training set are 0.138, 0.104, 0.143, and 0.102, respectively. In machine learning, a significant difference in accuracy between the training and test sets usually indicates overfitting. To prevent overfitting, this study selected CatBoost with a minor difference in the AUC between the training and test sets as the optimal model for the research.

**Table 2:** Comparison of Sensitivity Model Accuracy for Landslides in Northern Shaanxi.

Model	Accuracy	Precision	Recall	F1-Score	AUC_Test	AUC_Train
Random Forest	0.7379	0.7219	0.774	0.747	0.8217	0.9596
XGBoost	0.7414	0.7278	0.7713	0.7489	0.8265	0.9314
LightGBM	0.7533	0.7392	0.7828	0.7604	0.8302	0.9726
CatBoost	0.7485	0.7348	0.7775	0.7556	0.8307	0.9334

#### 4.2 Model selection for the southern Shaanxi region.

In the Qinba Mountainous Area of southern Shaanxi, where tectonic faults and complex lithology are prevalent, the landslide development pattern is highly nonlinear. This study also adopted a 7:3 split ratio for the dataset. It evaluated its applicability in a rocky, mountainous environment by comparing and analyzing the ROC curves of Random Forest, XGBoost, LightGBM, and CatBoost.

**Fig 7:**
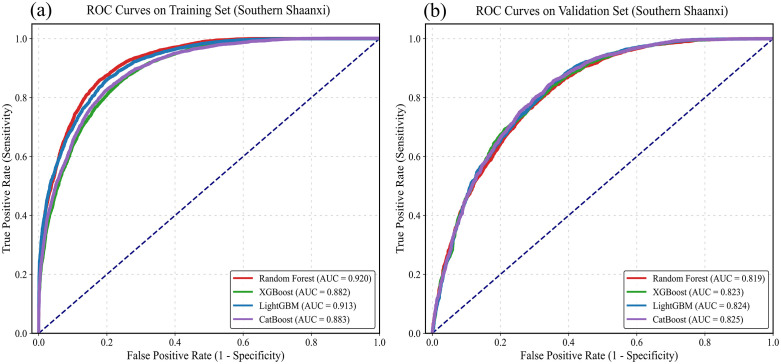
Augmentation values of ROC curves in Southern Shaanxi. (a) training set; (b) test set.

During the training phase, as shown in Fig 7a and Table 3, all four machine learning models demonstrated strong learning on the training set in southern Shaanxi. The AUC values of Random Forest, XGBoost, LightGBM, and CatBoost were 0.920, 0.882, 0.913, and 0.883, respectively. Its ROC curve shows the best predictive performance and accurately captures the control of tectonic zones and weak rock groups on landslides. As shown in Fig 7b, during the testing phase, 30% of the validation samples had AUC values of 0.812, 0.823, 0.824, and 0.825 for Random Forest, XGBoost, LightGBM, and CatBoost, respectively. The differences are 0.108, 0.059, 0.089, and 0.058, respectively. CatBoost has the strongest generalization ability among the four. In contrast, traditional integrated algorithms (such as RF) have shown some fluctuation in predictive performance when dealing with complex, random noise in the mountainous areas of southern Shaanxi.

**Table 3:** Comparison of Sensitivity Model Accuracy for Landslides in Southern Shaanxi.

Model	Accuracy	Precision	Recall	F1-Score	AUC_Test	AUC_Train
Random Forest	0.7384	0.7063	0.8159	0.7571	0.8186	0.9196
XGBoost	0.741	0.7135	0.8049	0.7565	0.8227	0.8815
LightGBM	0.7427	0.7169	0.8018	0.7570	0.824	0.9134
CatBoost	0.7483	0.7229	0.8049	0.7617	0.8252	0.8834

**Fig 8:**
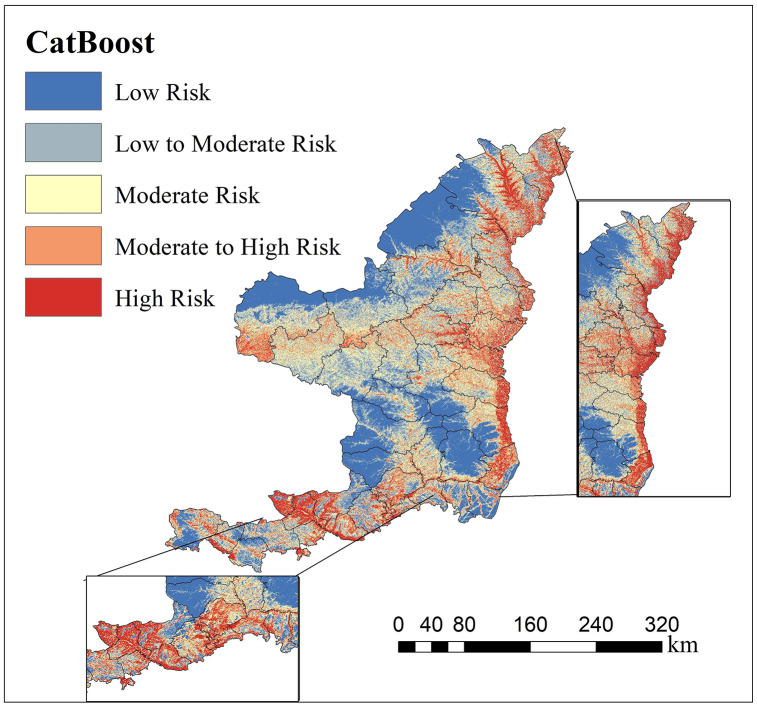
Prediction map of landslide-prone areas in Northern Shaanxi. The prediction map further categorizes the susceptibility into five levels (from low to high risk), revealing the landslide distribution patterns in the Northern Shaanxi loess hilly-gully regions.

Based on the model generalization results of northern and southern Shaanxi, CatBoost demonstrated the best generalization performance in both geomorphic regions (AUC = 0.8307 for the northern Shaanxi test set, AUC = 0.8252 for the southern Shaanxi test set), and the accuracy difference between the training set and the test set was the smallest, effectively suppressing overfitting. This indicates that the CatBoost algorithm has significant advantages in handling complex nonlinear relationships across terrains and applies to two distinct geological environments: the loess region and the bedrock mountainous area.

#### 4.3 Spatial prediction of cross-landform landslide susceptibility.

A 30 × 30 m grid was adopted as the evaluation unit, and the sample set was randomly split into a training set and a test set at an 7:3 ratio. The trained CatBoost models were applied to the northern and southern regions of Shaanxi to predict the spatial distribution of landslide-occurrence probability. Based on the predicted probability values, this study divides the [0,1] interval into five grades: Low, low-moderate, Moderate, moderate-high and High susceptibility areas.

As shown in Fig 8, the landslide susceptibility in the northern Shaanxi region presents significant spatial characteristics of “gully dependence” and “road network aggregation”. The high and medium-high-susceptibility areas mainly form strips or patches along the edges of ridges and mounds, steep slopes, and both sides of deep gullies on the Loess Plateau. The terrain in these areas is highly undulating, and the slopes are mainly in the critical range where landslides are prone to occur. More crucially, the distribution of areas with a higher susceptibility level closely matches that of the major transportation arteries (DTR) within these areas. This phenomenon profoundly reveals the extreme sensitivity of the landslides in northern Shaanxi to human engineering activities – high-intensity human disturbances such as “cutting slopes for road construction” and “building houses by cutting slopes” have become the dominant external forces driving the occurrence of loess landslides. Its spatial effect even surpasses the controlling role of natural terrain factors. The spatial manifestation of this “human-land” contradiction provides a clear direction for formulating differentiated disaster-prevention strategies in the northern Shaanxi region: risk control should prioritize monitoring slope stability and engineering reinforcement along roads and in residential areas.

**Fig 9:**
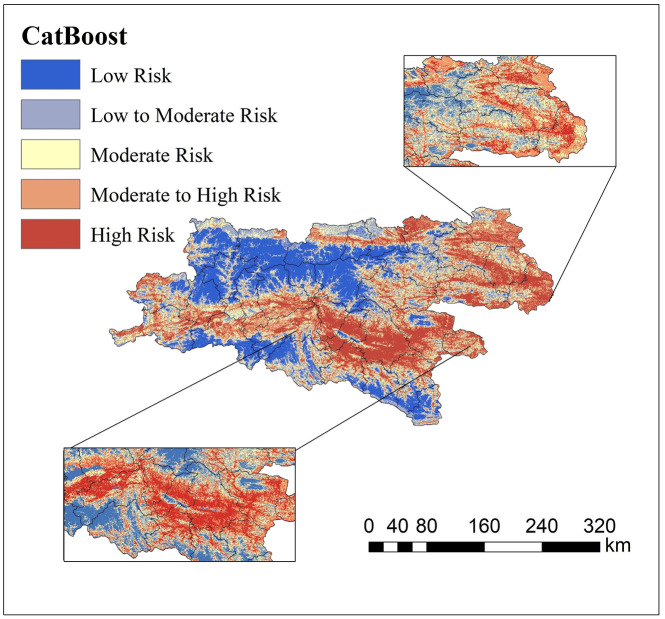
Prediction map of landslide-prone areas in Southern Shaanxi. The prediction map further categorizes the susceptibility into five levels (from low to high risk), revealing the landslide distribution patterns in the Southern Shaanxi mountainous regions.

As shown in Fig 9, the distribution of landslide susceptibility in the southern Shaanxi region reflects strong characteristics of “geological structural constraints” and “water system directionality”. The high- and medium-high-susceptibility areas mainly extend along the tectonic fault zone (DTF) between the Qinling-Bashan Mountains and the tributary valleys of major rivers, including the Han River and the Jialing River. Their distribution patterns are relatively continuous and are significantly controlled by the macroscopic geological framework. Unlike the “discrete patchy” distribution in northern Shaanxi, the prone spatial pattern in southern Shaanxi shows distinct “band-like continuity”, reflecting the essential feature that regional tectonic lines control the development of landslides in rocky mountainous areas. Most importantly, the distribution of susceptibility is closely related to the Lithology of local strata. In the distribution areas of brittle or weak rock groups, such as metamorphic and clastic rocks, the susceptibility grade rapidly transitions from “medium” to “high”, reflecting the fundamental control of rock mass structure on slope stability. This discovery indicates that the focus of disaster prevention in the southern Shaanxi region should be on systematic investigation, monitoring, and early warning of the distribution areas of structural fracture zones and weak rock groups.

By comparing the spatial prediction results for the two places, a significant cross-geomorphic differentiation pattern emerges. However, the CatBoost model’s prediction process lacks intuitive interpretability. It is difficult to answer key questions directly, such as “Why is this area determined to be high-risk?” and “What are the differences in disaster-causing logic among different landform areas?” This “black box” feature not only undermines the model’s credibility in disaster-prevention decision-making but also hinders a deeper understanding of the physical mechanisms of landslides. Therefore, this paper subsequently introduces the SHAP interpretation framework to conduct global attribution and local decomposition of the model’s predictions, to reveal the differentiating factors driving landslide occurrence in northern and southern Shaanxi and their nonlinear action thresholds [39].

#### 4.4 Interpretable analysis of cross-landform disaster-causing mechanisms.

##### 4.4.1 Comparison of global factor importance.

**Fig 10:**
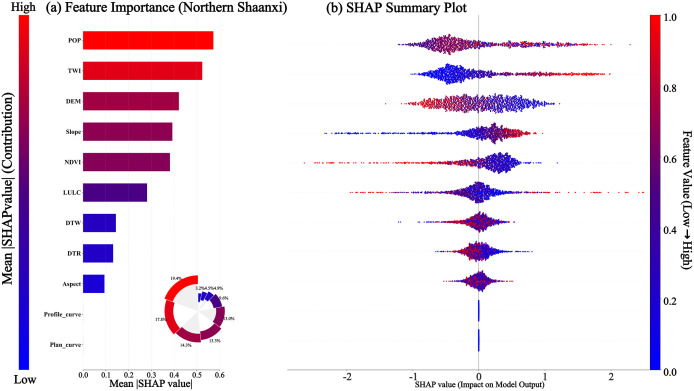
SHAP Analysis Map of Northern Shaanxi Region. **(a)** The sub-figure illustrates the global feature importance for landslide prediction in Northern Shaanxi, with population density (POP) and Topographic Wetness Index (TWI) identified as the most influential factors. **(b)** The summary plot reveals the specific impact of feature values: the color represents the feature value (red for high, blue for low), and the horizontal axis indicates the contribution to the landslide probability.

**Fig 11:**
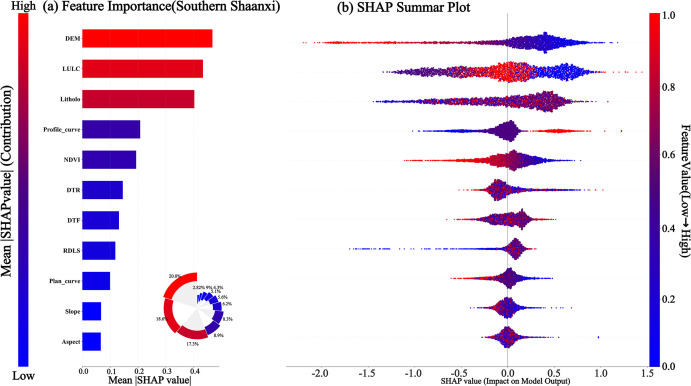
SHAP Analysis Map of Southern Shaanxi Region. **(a)** The sub-figure displays the global importance ranking of evaluation factors based on the mean absolute SHAP values, identifying DEM, LULC, and Lithology as the dominant factors. **(b)** The summary plot further reveals the direction of influence: colors represent feature values (red for high, blue for low), and SHAP values greater than 0 indicate an increased probability of landslide occurrence.

The global attribution analysis of the SHAP framework directly demonstrates the differences in decision-making logic between the CatBoost model in the loess region of northern Shaanxi and the Qinba Mountain Area of southern Shaanxi. It can be seen from Figs 10a and 11a that the population density POP in the northern Shaanxi region ranks first with a contribution rate of 19.4%, followed closely by the topographic humidity index TWI with 17.8%. The combined contribution of the two exceeds 37%, while the elevation DEM ranks only third. In the southern Shaanxi region, a completely different power distribution is presented. Elevation DEM dominates model decision-making, with an absolute advantage of 20%; land use LULC and stratum lithology Litholo form secondary driving clusters with 18.6% and 17.3%, respectively; and the human activity factor DTR is marginalized at 6.2%. In addition, the NDVI contribution rates of the Northern Shaanxi region and the Southern Shaanxi region are 13.05% and 8.3% respectively. Further analysis of the tail characteristics of factor ranking shows that the Profile_curve ranked in the top five, with a contribution rate of 8.9% in the southern Shaanxi region. In contrast, the model almost ignored it in northern Shaanxi.

From the above, it can be seen that the result presented by the SHAP global analysis challenges the implicit assumption of “factor universality” in the traditional landslide susceptibility evaluation. The same set of input variables has undergone a thorough reorganization of weights in different geological environments, which means that the model transfer across landforms not only requires parameter adjustment but also the structural reconstruction of the factor system. The fact that the CatBoost model maintains high accuracy in both terrain regions precisely masks this profound difference in internal decision-making logic – the tension between the high predictive performance of the black box and the low transparency of the mechanism is precisely the fundamental motivation for introducing SHAP for interpretability analysis in this study.

##### 4.4.2 Single-factor nonlinear response and physical threshold recognition.

Figs 10b and 11b present SHAP summary plots in the form of Bee Swarm plots, which are used to visually analyze the relationship between the values of disaster-causing factors and the probability of landslides. Fig 10b shows that population density (POP), terrain moisture index, elevation, Slope, vegetation coverage, and land use have the most significant impact on landslide occurrence in northern Shaanxi, among which POP shows the strongest positive correlation. The red scattered dots are concentrated on the right side. indicating that, as population density increases, the risk of landslides has significantly increased due to the intensification of human life and production activities. The terrain moisture index (TWI), elevation (DEM), and Slope (Slope) all show a positive correlation. In contrast, vegetation coverage (NDVI), distance to water systems (DTW), and distance to roads (DTR) are negatively correlated. The smaller the characteristic values, the higher the probability of landslides.

It can be seen from Fig 11b that elevation (DEM), land use (LULC), lithology of strata (Litholo), profile curvature (Profile_curve), vegetation coverage (NDVI), and distance from road (DTR) in the southern Shaanxi region have the most significant influence on the occurrence of landslides in the southern Shaanxi region, among which elevation (DEM) is positively correlated. The red scattered points (high values) are significantly concentrated in the right area. This indicates that in the Qinba Mountainous Area, the higher the altitude, the greater the potential energy of slope development, and the risk of landslides increases significantly accordingly. On the contrary, both vegetation coverage (NDVI) and distance from road (DTR) are negatively correlated. The smaller the characteristic value, the higher the probability of landslide occurrence.

**Fig 12:**
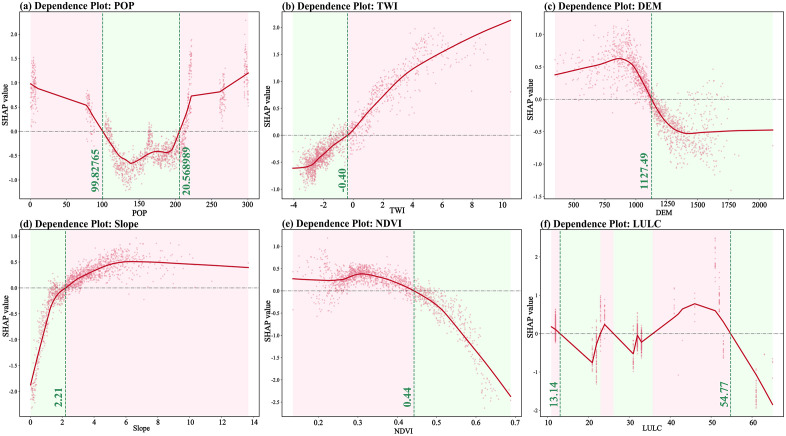
Single-factor dependency map of the Northern Shaanxi region. **(a)** Dependence Polt: POP; **(b)** Dependence Polt: TWI; **(c)** Dependence Polt: DEM; **(d)** Dependence Polt: Slope; **(e)** Dependence Polt: NDVI; **(f)** Dependence Polt: LULC. The red solid lines represent the marginal effects of evaluation factors on landslide susceptibility, while the pink dots indicate sample distribution. Green dashed lines and shaded areas denote the critical thresholds for each factor.

To deeply analyze the quantitative relationship between the characteristic values of disaster-causing factors and landslide prediction results, this study introduces a single-factor Dependence Plot (SHAP Dependence Plot) for global interpretation. For the northern Shaanxi region, it can be seen from Fig 12a that when the population density (POP) crossed the critical threshold of approximately 99.83 people/km^2^, the SHAP value rapidly turned from negative to positive and continued to rise, indicating that the strong anthropogenic disturbance significantly exceeded the self-regulating capacity of the loess environment and became the core external inducible driving the occurrence of landslides. The terrain moisture index (TWI) in Fig 12b reveals that when TWI reaches approximately −0.40, its contribution to landslide prediction remains positive. In Fig 12c, at approximately 1127.49 m, the SHAP value shows a significant positive contribution. Its contribution to landslides increases with increasing altitude. In Fig 12d, when the Slope exceeds approximately 2.21°, the dependency graph shows a powerful monotonically increasing feature. Once the slope threshold is crossed, the SHAP value switches from negative to positive, increasing the likelihood of landslides. Fig 12e shows that when vegetation coverage reaches approximately 0.44, NDVI exhibits a significant negative response. Below the threshold of 0.44 (in low-vegetation areas), the SHAP value is positive, indicating a higher likelihood of landslides. When the NDVI exceeds 0.44, the SHAP value drops below zero, which inhibits the occurrence of the landslide. From Fig 12f, when the land use characteristic values are around 13.14 and 54.77, there is a positive correlation with landslides. As a classification factor, its SHAP value shows noticeable positive fluctuations within these specific value ranges.

**Fig 13:**
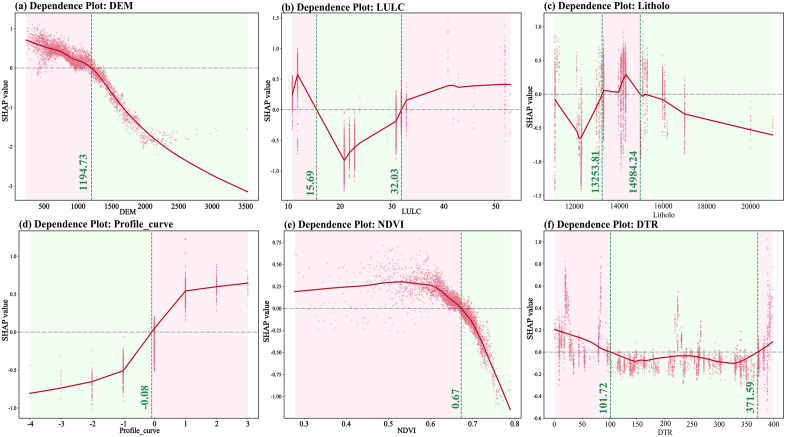
Single-factor dependency map of the Southern Shaanxi region. **(a)** Dependence Polt: DEM; **(b)** Dependence Polt: LULC; **(c)** Dependence Polt: Litholo; **(d)** Dependence Polt: Profile_curve; **(e)** Dependence Polt: NDVI; **(f)** Dependence Polt: DTR. The red solid lines represent the marginal effects of evaluation factors on landslide susceptibility, while the pink dots indicate sample distribution. Green dashed lines and shaded areas denote the critical thresholds for each factor.

In the southern Shaanxi region, as shown in Fig 13a, when the elevation (DEM) height exceeds approximately 1194.73 meters, the SHAP value turns from negative to positive. It provides a large amount of gravitational potential energy, increasing the likelihood of landslides. reveals the nonlinear discrete distribution of land use (LULC). When the eigenvalues are around 15.69 and 32.03 (Fig 13B), the probability of landslide occurrence increases significantly, revealing differentiated risk regulation by land cover. Fig 13c shows a distinct interval effect. When the Lithology characteristic values are within the range of 13,253.81 to 14,984.24, the probability of landslide occurrence significantly increases. Within this numerical range, a substantial positive SHAP contribution is observed, reflecting the fundamental control that rock mass structure exerts on slope stability. Fig 13d shows that when the curvature changes from negative (concave Slope) to positive (convex slope), the SHAP value crosses zero, significantly increasing the probability of landslide occurrence. As shown in Fig 13e, when the NDVI is below 0.67, the SHAP value is positive, indicating an increased probability of landslide occurrence. Once it exceeds this threshold, the SHAP value rapidly drops below zero, and the probability of landslide occurrence increases. Fig 13f reveals that when the distance from the road is within the range of 101.72m to 371.59m, the probability of landslides occurring is significantly reduced.

##### 4.4.3 Analysis of cross-geomorphic factor interaction effects.

**Fig 14:**
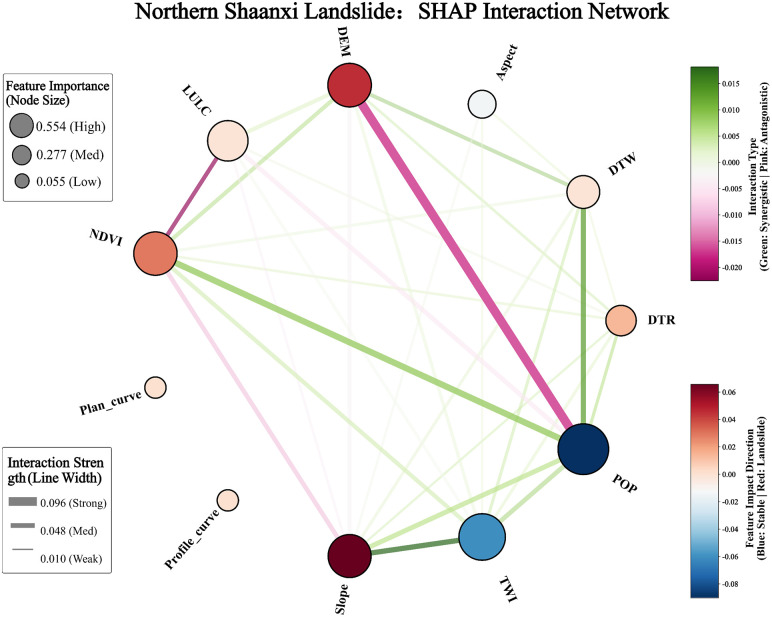
Correlation Analysis of Evaluation Factors in Northern Shaanxi Region. Node size represents the feature importance, and the thickness of the lines indicates the interaction strength between factors. Line colors denote the interaction type (green for synergistic and pink for antagonistic), while node colors reflect the impact direction of each factor on landslide susceptibility (red for increasing risk and blue for decreasing risk).

**Fig 15:**
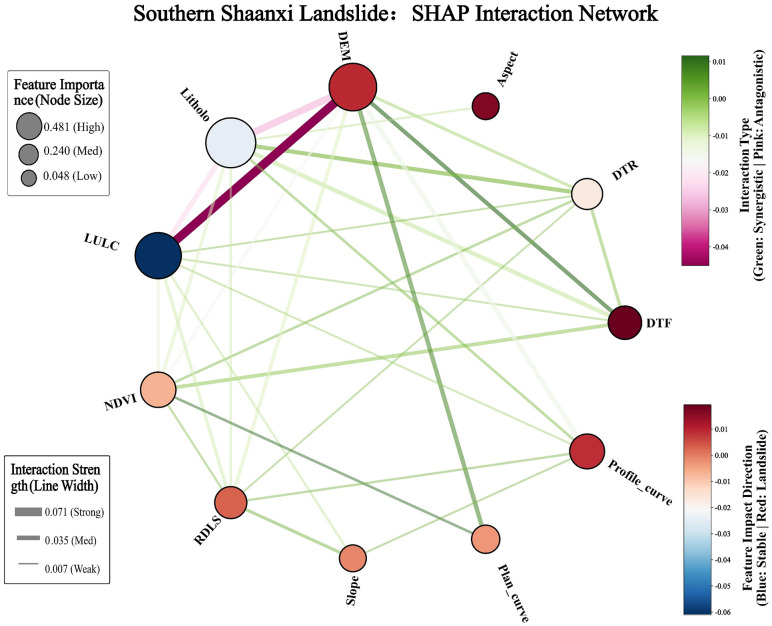
Correlation Analysis of Evaluation Factors in Southern Shaanxi Region. Node size represents the feature importance, and the thickness of the lines indicates the interaction strength between factors. Line colors denote the interaction type (green for synergistic and pink for antagonistic), while node colors reflect the impact direction of each factor on landslide susceptibility (red for increasing risk and blue for decreasing risk).

Although single-factor analysis reveals the marginal contribution of each variable to risk, landslides in natural environments are often driven by multiple factors. Based on the identification of the single-factor dependence law and the physical thresholds of landslide evaluation factors in the northern and southern Shaanxi regions, further exploration of the interactions and Coupling Effects among various factors is crucial for analyzing the complex landslide mechanism. For this purpose, this study introduces the SHAP Interaction Network (Figs 14 and 15).

Fig 14 shows the SHAP interaction network of landslide evaluation factors in the northern Shaanxi region. In the figure, the red nodes in the DEM (Elevation) indicate that this factor has a positive effect on the model. In contrast, the dark blue nodes for population density (POP) indicate a significant inhibitory effect. From the figure, it can be continuously observed that there are significant thick and solid lines connecting the population density (POP), the terrain moisture index (TWI), the Slope (Slope) and the distance from the hydrological level (DTW), indicating that these four factors have a significant synergistic effect on the landslides in northern Shaanxi. The maximum interaction intensity in the figure is 0.096, occurring between the elevation (DEM) and population density (POP) factors, indicating a strong antagonistic effect. Meanwhile, land use (LULC) and vegetation utilization rate (NDVI) also have relatively apparent antagonistic effects [40].

Fig 15 presents the shape-interaction diagram of landslide evaluation factors in the southern Shaanxi region. In the figure, the red nodes representing elevation (DEM) have a positive effect on the model’s predictions. The large blue edge nodes with vegetation coverage rate (NDVI) in the figure indicate that they mainly play an independent inhibitory role. From the connection lines in the figure, there is a relatively noticeable synergistic effect among elevation (DEM), distance from fault (DTF), and planar curvature (Plan_curve). On the contrary, there is an apparent antagonistic relationship between elevation (DEM) and LULC (land use), with an interaction intensity of 0.071. Meanwhile, elevation (DEM), lithology (Litholo), and land use (LULC) also have relatively apparent antagonistic effects.

From the above, it can be seen that the strongest interaction in northern Shaanxi occurs between DEM and POP (0.096), indicating an antagonistic effect and reflecting the spatial differentiation between sparse populations in high-altitude areas and dense human activities in low-altitude gully areas. The strongest interaction in the southern Shaanxi region occurred between DEM and LULC (0.071), indicating an antagonistic effect. revealing limited land use in high-altitude areas and concentrated human activities in low-altitude areas. The differences in the dominant interaction patterns between the two regions reflect the logic of disaster causation: “human-water” coupling in northern Shaanxi and “structure-topography” control in southern Shaanxi.

## Discussion

### 5.1 Cross-geomorphic landslide vulnerability prediction

The spatial prediction results for Northern and Southern Shaanxi exhibit pronounced cross-geomorphic differentiation. Specifically, landslide susceptibility in Northern Shaanxi manifests an anthropogenically driven spatial pattern, where high-susceptibility zones are closely coupled with population and road densities, displaying distinct point-and-line distribution characteristics. In contrast, Southern Shaanxi presents a tectonic-controlled spatial configuration, with high-susceptibility areas distributed in belt-like and planar patterns along fault zones and drainage systems, showing high congruence with geological structural lines. This morphological divergence fundamentally reflects the essential differences in disaster-triggering mechanisms between the two regions: human-water coupling prevails in the loess region, whereas tectonic-topographic control dominates the rocky mountainous region. The statistical progression of increasing landslide density from low to high susceptibility zones validates the capability of the CatBoost model to accurately capture the landslide development modes of both regions. Furthermore, grid-based machine learning models also prove effective for predicting landslide susceptibility across heterogeneous geomorphic environments.

### 5.2 Analysis of cross-geomorphic landslide influencing factors

A comparison between Fig 10 and Fig 11 reveals that the spatial reorganization of factor importance across the two regions is far more than mere numerical fluctuation; rather, it reflects fundamentally distinct instability dynamics inherent to these geomorphic units. The causative logic of the loess plateau in Northern Shaanxi exhibits a pronounced sensitivity to anthropogenic disturbances. The high contribution of POP, coupled with the synergistic effect of TWI, suggests that landslide occurrences are driven by the spatiotemporal coupling between human engineering activities and moisture infiltration. Specifically, once slope-cutting disrupts the structural integrity of the loess, hydrological variables become the decisive triggers for slope failure. This mechanism stands in stark contrast to the rocky mountainous areas of Southern Shaanxi, where the dominance of DEM and lithology underscores the fundamental control exerted by gravitational potential energy and rock mass shear strength. In the latter, human activities serve merely as modifying variables, incapable of overriding the primary constraints of geological structure and topographic energy.

Furthermore, both NDVI and LULC contributed at moderate levels in the two geomorphic regions. This seemingly consistent appearance actually conceals completely different ecological-disaster coupling mechanisms. The 13.05% contribution rate of NDVI in the northern Shaanxi region reflects the mechanical reinforcement effect of vegetation root systems on shallow loess sliding, and its failure threshold is closely related to TWI. The 8.3% contribution rate of NDVI in the southern Shaanxi region is more evident in the stabilizing effect of vegetation coverage on the weathering products of bedrock, and its effectiveness is controlled by the thickness of surface materials, as determined by Lithology. If the NDVI contributions of the two landform areas are directly compared, it will lead to a simplified understanding that “the richer the vegetation, the lower the risk of landslides”, ignoring the matching relationship between vegetation types, root depth and geological matrix [41].

Further analysis of the tail characteristics of factor ranking shows that the Profile_curve ranked in the top five, with a contribution rate of 8.9% in the southern Shaanxi region. In contrast, the model almost ignored it in northern Shaanxi. This difference precisely confirms the completely different slope surface processes of the homogeneous overburden layer of loess and the bedrock structural surface: the slope shape changes of the loess mound-shaped landforms in northern Shaanxi are smoothed by the homogeneous soil layer, and the macroscopic topographic indicators absorb the influence of curvature on stability. The bedrock slopes in the southern Shaanxi region are extremely sensitive to local curvature changes. The stress concentration at the convex slope and the superposition of structural joints become the controlling factors. This weight distribution in the model’s automatic learning is actually a data-based confirmation of the differences in the slope processes of the two geomorphic units.

### 5.3 Interaction analysis of cross-geomorphic landslide influencing factors

In Northern Shaanxi, DEM and POP exhibit a pronounced antagonistic effect. This highlights the significant impact of the unique “gully-ridge” vertical functional zoning in the Loess Plateau on the underlying disaster logic. In low-altitude gully regions, high population density and intensive engineering activities disrupt the natural equilibrium of loess slopes, establishing “human disturbance” as the primary driver of landslides. Conversely, as elevation increases into the ridge and mound areas, the extremely fragmented terrain restricts human activity, causing the landslide mechanism to shift toward dominance by natural factors. This spatial “trade-off” indicates that the landslide mechanism in Northern Shaanxi undergoes a fundamental transition from anthropogenic induction to natural regulation along the vertical profile. A pronounced antagonistic effect is observed between DEM and LULC in Southern Shaanxi. This reflects the fundamental constraints imposed by rugged topography on the intensity of land development. In high-altitude bedrock mountainous regions, land-use patterns are relatively homogeneous, consisting primarily of forestland or bare rock, where landslide occurrence is predominantly governed by high gravitational potential energy and tectonic fracture zones. Conversely, in low-altitude areas surrounding the Hanzhong or Ankang basins, land-use characteristics become increasingly complex. Here, anthropogenic land transformations tend to mask the underlying controlling effects of natural topographic features.

### 5.4 Limitations of the study and future research

The evaluation factors employed in this study are primarily conventional static variables, thereby neglecting the triggering effects of short-term extreme rainfall events and seismic activities. The absence of analysis regarding such extreme events may limit the model’s predictive performance in short-term early warning scenarios. Furthermore, although the 30m resolution is widely accepted for regional-scale mapping, it remains relatively coarse and may fail to precisely capture micro-topographic features or small-scale loess landslides that are frequently observed in fragmented gully regions.

Future research could utilize higher-resolution data to identify more subtle terrain characteristics and the occurrence of minor landslides. Additionally, by incorporating landslide prediction under extreme triggering conditions, the current static vulnerability mapping can be effectively evolved into a more comprehensive dynamic hazard forecasting system.

## Conclusion

(1) The CatBoost model demonstrated optimal generalization performance in both the loess region of northern Shaanxi and the rocky mountainous area of southern Shaanxi. The AUCs for the test sets were 0.8307 and 0.8252, respectively, with the smallest difference in accuracy between the training and test sets. This effectively suppressed overfitting and verified the algorithm’s robust prediction ability in complex nonlinear environments across terrains.
(2) The spatial pattern of landslide susceptibility shows significant cross-geomorphic differentiation: in northern Shaanxi, it is characterized by a discrete patch distribution of “gully dependence” and “road network aggregation”, with human engineering activities becoming the dominant driving force. In southern Shaanxi, there is a continuous band-like distribution along the tectonic fault zone and water system, which is significantly controlled by geological structure and lithology.
(3) SHAP global attribution reveals the completely different disaster-causing logics of the two landform regions: In northern Shaanxi, the contribution rate of population density of 19.4% and terrain moisture index of 17.8% presents the “human-water” coupled driving characteristics, while in southern Shaanxi, the contribution rate of elevation of 20% and lithology of 17.3% presents the “structure-terrain” control characteristics. The same factor system has undergone a complete reorganization of weights in different geological environments.
(4) The single-factor dependency map accurately identified the physical threshold differences across landforms: the critical value of population density in northern Shaanxi was 99.83 people/km^2^, the slope threshold was 2.21°, and the elevation threshold in southern Shaanxi was 1194.73 m. Moreover, the inversion thresholds of the NDVI action direction in the two regions were 0.44 and 0.67, respectively. The SHAP interaction network further quantified the antagonistic effects of DEM and POP in northern Shaanxi (0.096) and DEM and LULC in southern Shaanxi (0.071), revealing the essential differences between “human-water” coupling and “construction-terrain” control.
(5) As indicated by this study, for landslide prevention in Northern Shaanxi, slope-cutting for housing and road construction should be strictly restricted within these critical threshold areas. Comprehensive drainage engineering should be implemented, with a particular emphasis on the interception and diversion of seepage from trailing-edge fissures to reduce the triggering effect of water on disturbed loess slopes. Simultaneously, as landslides in Southern Shaanxi are predominantly induced by heavy rainfall, slopes near tectonic fracture zones should be the focus of inspections during the rainy season. For the high-susceptibility areas common in this region, a real-time hierarchical early warning mechanism based on rainfall thresholds should be established.
